# Performance of the rapid triage conducted by nurses at the emergency entrance*

**DOI:** 10.1590/1518-8345.3467.3378

**Published:** 2020-10-19

**Authors:** Bruna Roberta Siqueira Moura, Lilia de Souza Nogueira

**Affiliations:** 1 Universidade de São Paulo, Hospital Universitário, Pronto Socorro Adulto, São Paulo, SP, Brazil.; 2 Universidade de São Paulo, Escola de Enfermagem, São Paulo, SP, Brazil.

**Keywords:** Triage, Patient Acuity, Emergencies, Emergency Medical Services, Nursing, Efficiency, Triagem, Gravidade do Paciente, Emergências, Serviços Médicos de Emergência, Enfermagem, Eficiência, Triaje, Gravedad del Paciente, Urgencias Médicas, Servicios Médicos de Urgencia, Enfermería, Eficiencia

## Abstract

**Objective:**

to compare the performance of the rapid triage conducted by nurses at the emergency entrance and of the Manchester Triage System (MTS) in identifying the priority level of care for patients with spontaneous demand and predicting variables related to hospitalization.

**Method:**

a cross-sectional study carried out in an Emergency Department (ED) of a university hospital in São Paulo. The priority levels established in the rapid triage performed by nurses were high priority (patients of spontaneous demand directed to the emergency room) or low priority (those referred to the institution’s usual flow). Diagnostic accuracy measures were calculated to assess the performance of the indexes.

**Results:**

of the 173 patients (52.0% female, with mean age of 60.4 ± 21.2 years old) evaluated, it was observed that rapid triage was more inclusive for high priority and had better sensitivity and worse specificity than the MTS. The probability of non-severe patients being admitted to the emergency observation unit was lower due to the rapid triage. For the prediction of the other variables, the systems presented unsatisfactory results.

**Conclusion:**

the nurses overestimated the classification of patients as high priority, and rapid triage performed better than MTS in predicting admission to the emergency observation unit.

## Abstract (pt)

**Objetivo:**

comparar o desempenho da triagem rápida realizada pelos enfermeiros na porta de emergência e do Sistema Manchester de Classificação de Risco (SMCR) na identificação do nível de prioridade de atendimento dos pacientes de demanda espontânea e predição de variáveis relacionadas à internação hospitalar.

**Método:**

estudo transversal realizado em um Pronto Socorro (PS) de hospital universitário em São Paulo. Os níveis de prioridade estabelecidos na triagem rápida realizada pelos enfermeiros foram alta prioridade (pacientes de demanda espontânea direcionados à sala de emergência) ou baixa prioridade (aqueles encaminhados ao fluxo habitual da instituição). Medidas de acurácia diagnóstica foram calculadas para avaliar o desempenho dos índices.

**Resultados:**

dos 173 pacientes (52,0% sexo feminino; idade média 60,4±21,2 anos) avaliados, observou-se que a triagem rápida foi mais inclusiva para alta prioridade e apresentou melhor sensibilidade e pior especificidade do que o SMCR. A probabilidade de pacientes não graves serem admitidos na observação da emergência foi menor pela triagem rápida. Para a predição das outras variáveis, os sistemas apresentaram resultados insatisfatórios.

**Conclusão:**

os enfermeiros superestimaram a classificação de pacientes como alta prioridade e a triagem rápida obteve melhor desempenho que o SMCR na predição de admissão na sala de observação do PS.

## Abstract (es)

**Objetivo:**

comparar el desempeño del triaje rápido realizado por el personal de enfermería en la puerta de emergencias y del *Manchester Triage System* (MTS) al identificar el nivel de prioridad de atención a los pacientes de demanda espontánea y la predicción de las variables relacionadas con la internación.

**Método:**

estudio transversal realizado en un Servicio de Emergencias (SE) de un hospital universitario de São Paulo. Los niveles de prioridad establecidos en el triaje rápido realizado por los enfermeros fueron de alta prioridad (pacientes de demanda espontánea dirigidos a la sala de emergencias) o de baja prioridad (los remitidos al flujo habitual de la institución). Se calcularon medidas de exactitud diagnóstica para evaluar el desempeño de los índices.

**Resultados:**

de los 173 pacientes (52,0% mujeres; edad promedio 60,4±21,2 años) evaluados, se observó que el triaje rápido fue más inclusivo en casos de alta prioridad y presentó mejor sensibilidad y peor especificidad que el MTS. La probabilidad de que los pacientes no graves fueran admitidos en el área de observación del servicio de emergencias fue menor gracias al procedimiento de triaje rápido. En cuanto a la predicción de las demás variables, los sistemas presentaron resultados no satisfactorios.

**Conclusión:**

los enfermeros sobrestimaron la clasificación de los pacientes como alta prioridad y el triaje rápido obtuvo un mejor desempeño que el MTS para predecir el ingreso en la sala de observación del SE.

## Introduction

Overcrowding in emergency services is a reality in many institutions. In this scenario, the triage emerged as a tool to optimize care in emergencies and to identify patients who need to have priority in care and treatment, through a dynamic assessment process^(^ 1 ^-^ 2 ^)^.

Among the different triage systems applied in emergency services, the Manchester Triage System (MTS)^(^ 3 ^)^ stands out as one of the most used in Brazilian institutions. The MTS is based on the identification of the patient’s main complaint and establishes, through decision flowcharts and discriminators, the maximum time for the first medical assessment^(^ 3 ^)^. Thus, patients classified as red (emergency) by the MTS need immediate care, as orange (very urgent) in up to 10 minutes, as yellow (urgent) in a maximum of 60 minutes, as green (not very urgent), as and blue (not urgent) in up between 120 and 240 minutes, respectively^(^ 3 ^)^.

Despite the proven importance of triage in the organization of the emergency services, the waiting time between the opening of the service record and the triage routine can vary according to the demand of the moment, making it possible to wait in queues, which, for some patients, it means serious health problems due to the delay in starting their treatments^(^ 2 ^)^. In addition, a study that analyzed patients classified in the red category according to the MTS identified in the sample the mean time between arrival at the institution and the end of the 8-minute classification, which may represent valuable time spent for this type of patient^(^ 1 ^)^.

Still in the case of patients classified in the red category, research shows that, in some emergency services, critically ill patients are generally seen even before opening the hospital registration form, and that triage routine is performed retroactively, after the patient’s clinical stabilization^(^ 1 ^,^ 4 ^-^ 5 ^)^.

Therefore, it is noticeable that the implementation of a triage protocol does not guarantee care at the recommended times, thus it is essential to organize management and assistance flows that speed up the patients’ access to the service, care and treatment at the appropriate times according to their level of severity^(^ 5 ^)^. This is the case of high priority patients (emergency and very urgent), for example, who often need a quick professional evaluation (in this study called “rapid triage”), still at the hospital’s emergency entrance, so that it is possible to early detect their severity and proceed to the immediate care in the emergency room.

In the present study, rapid triage, applied only to patients who arrive at the service reporting severity at the emergency entrance, is performed empirically, without protocols and/or triage systems, that is, the nurse makes a quick assessment of the patients’ general condition and complaint, still in the transportation vehicle, to determine if they are facing an emergency and need immediate care (referral to the emergency room) or if they can follow the normal flow of the institution (opening the hospital registration form and waiting for the triage routine in a non-critical sector).

It is worth noting that the objective of rapid triage at the emergency entrance is to identify, among patients of spontaneous demand, those with a potential life risk and, therefore, who require immediate decision-making by the health professional (physician or nurse) working in the emergency service, based on clinical data, subjective information and previous experience^(^ 6 ^)^, in addition to the use of cognitive and intuitive processes. This situation differs from those patients referred to the hospital by pre-hospital service vehicles or private ambulances, as they are assisted by a health professional and have already received initial care.

Finally, the correct identification of patients with high priority through rapid triage increases the chances of survival. On the other hand, the identification of low priority patients (urgent, little urgent or non-urgent) avoids overcrowding in the emergency sector, preventing human and material resources from being diverted to the care of those without real serious conditions and that could be assessed in less critical sectors^(^ 2 ^)^.

In view of the above, there was concern about the rapid triage performed by nurses in cases of patients coming from spontaneous demand and who arrive at the emergency entrance referring severity. Some questions guided this concern: If the same patients were screened by the MTS protocol, would they have the same classification? What is the performance of rapid triage compared to the MTS in predicting different variables related to patients’ hospital admission?

In this sense, the objective of this research was to compare the performance of the rapid triage performed by nurses at the emergency entrance and of the MTS in identifying the priority level (high or low) of care for patients with spontaneous demand and the prediction of variables related to hospitalization.

## Method

This is an observational, descriptive, and cross-sectional study with a quantitative approach developed in the adult Emergency Department (ED) of a secondary level university located in the city of São Paulo. The hospital serves patients with spontaneous demand, in addition to those referred by the pre-hospital service or from the public health network. Medical clinic, general surgery, orthopedics, otorhinolaryngology, bucomaxillofacial, gynecology, and obstetrics are the medical specialties present at the institution.

As for the hospital’s flow of care, patients seeking urgency and emergency services need to retrieve a password to open the hospital registration form at the reception. After this procedure, they wait in the waiting room (non-critical sector) for the triage procedure, which is performed by nurses qualified according to the MTS protocol. In the case of patients who come to the service due to spontaneous demand reporting severity at the emergency entrance, rapid triage is performed by the emergency room nurse and, if the patient is classified as high priority, medical care is immediately started in the critical sector and the hospital registration form is subsequently opened by the family member or companion. It is noteworthy that all the nurses of the institution’s ED have training on triage routine according to the MTS protocol.

The sample, for convenience, was composed of the evaluations (triage), carried out by the nurses of the ED, of patients aged 18 years old or over, coming from spontaneous demand, brought in private vehicles, and who arrived at the emergency entrance referring severity from May 1^st^ to December 13^th^, 2017 and at the time when the MTS is applied in the hospital (from 7 am to 7 pm).

Patients with obstetric complications or in labor were excluded from the research because, in these cases, they are referred directly to the obstetric emergency room, not being evaluated by the adult ED nurse.

To characterize the patients, the gender, age, number and type of comorbidities, clinic responsible for the first medical care, and medical diagnosis at discharge variables were analyzed. The priority level assigned by nurses after the rapid triage was identified as high priority (patients referred to the emergency room, trauma room, or emergency observation unit) or low priority (patients referred to the institution’s normal triage flow). For the MTS^(^ 7 ^)^, the priority categories were defined as high (red or orange colors) or low (yellow, green or blue colors) priority, determined according to the flowchart and discriminator identified from the main complaint of the patient or family member.

Regarding the hospital admission variables, the patient’s admission in the emergency observation unit, the length of hospital stay, admission to the Intensive Care Unit (ICU), and the condition when leaving the hospital (survivor or non-survivor) were analyzed.

For data collection, three instruments developed by the researchers were used and submitted to a pre-test for a period of 15 days prior to the beginning of data collection. At the end of the pre-test, no changes were necessary to the instruments initially proposed.

The first instrument, called “Rapid triage form”, consisted of the following information: date and time of triage, patient data (date of birth and gender), hospital record number, main complaint referred by the patient, signs and symptoms identified by the nurse according to the categories of breathing/ventilation, pulse, neurological dysfunction, perfusion, pain, hemorrhages, injuries and deformities, in addition to the referral given to the patient: high or low priority. Information on gender, signs and symptoms and referral given to the patient were made available in the checklist format. In addition, the instrument contained additional spaces for the descriptive insertion of the patient’s main complaint and other signs and symptoms not covered in the categories described above.

The second instrument, called “Triage Form adapted from the Manchester System”, was used to record data pertinent to triage performed by the researcher according to the MTS. This instrument included information on the patient’s hospital record, date and time of the triage, presentation of the situation/complaint and the MTS data (flowchart, discriminator, vital signs, and priority level assigned according to the system).

Finally, the third instrument, the “Form for characterizing the evolution of patients”, was used to collect data regarding the outcome of patients who were evaluated during the rapid triage. The instrument contained data on the number of the hospital record, comorbidities presented by the patient, the clinic responsible for the care, medical diagnosis at discharge, and length of hospital stay. In addition, in the checklist format, information about admission to the emergency observation unit, hospitalization in the ward, admission to the ICU and clinical outcome (discharge, death, evasion or transfer) of the patient were included in this instrument.

Data collection was carried out in two stages. In the first, information about the priority level assigned to spontaneous demand patients brought in private vehicles was identified from direct observation of the rapid triage performed by the nurses at the hospital’s emergency entrance. For this, at the end of each assessment, the researcher asked the triage nurse which classification was given to the patient (high or low priority). These data made it possible to fill in the “Rapid triage form” instrument. Concomitantly with rapid triage, but independently to ensure that there was no influence on the nurse’s evaluation, the researcher (also qualified by the MTS) classified these patients (high or low priority) by applying the MTS and by inserting the information in the “Triage Form adapted from the Manchester System”. In the second stage, the “Form for characterizing the evolution of patients” was filled out from the data retrieved from the medical records of patients related to emergency care, hospitalization, and clinical outcome. It is noteworthy that, for the data collection of this research, there was no change in the flow of patients in the institution.

The analysis of the performance of the rapid triage and of the MTS was assessed by identifying the sensitivity, specificity, accuracy, Positive Predictive Value (PPV), Negative Predictive Value (NPV), Positive Likelihood Ratio (PLR) and Negative Likelihood Ratio (NLR).

Due to the lack of clarity in the literature on a gold standard for rapid triage performed at the emergency entrance, the length of hospital over 24 hours, admission to the emergency observation unit, admission to the ICU, and death variables were tested for the real health condition (serious as high priority or not serious as low priority). The concepts of undertriage (patients classified as low priority and who have a real serious health condition) and overtriage (patients classified as high priority and who do not have a real serious health condition) were applied.

The study was approved by the Research Ethics Committee (opinion No. 1,969,864) and the Free and Informed Consent Form was obtained from the nurses and patients (or legal representatives) who participated in the research.

## Results

During the study period, 173 patients with spontaneous demand were evaluated and screened by nurses at the adult emergency entrance. Nine patients were evaluated on two different occasions during the study period (readmission cases at the institution), featuring two different evaluations and totaling 182 visits (evaluations).

Among the screened patients, the female gender (52.0%) prevailed, with a mean age of 60.4 (±21.2) years old. Systemic arterial hypertension (44.5%) was the most frequent comorbidity and patients had an approximate mean of two comorbidities.

Most of the patients’ visits (n=182) were clinical (72.0%), with the main diagnosis of hospital discharge being represented by symptoms, signs and abnormal clinical and laboratory findings, not classified elsewhere (24.2%). Most of the patients (67.6%) stayed less than 24 hours at the institution and/or were admitted to the emergency observation unit (56.0%). The survival rate was 86.8%.

**Figure 1:**
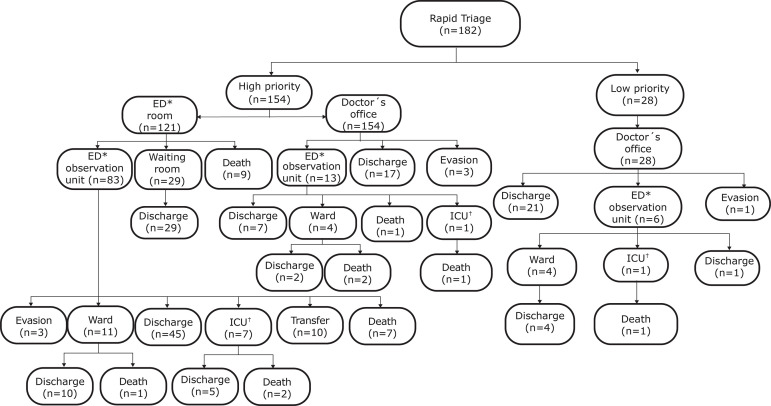
Flowchart of the referral and condition of patients leaving the institution after classification in the rapid triage. São Paulo, SP, Brazil, 2017 *ED = Emergency Department; ^†^ICU = Intensive Care Unit

The referral and condition of patients leaving the institution after classification in the rapid triage (high or low priority) are described in Figure 1. It is observed that, among the patients classified as high priority and who died (n=23), nine (39.2%) died during the first care in the emergency room. The remaining deaths (n=14) occurred in the emergency observation unit (34.8%), in the ward (13.0%), and in the ICU (13.0%). A patient classified as low priority by the nurse in the rapid triage died later in the ICU.

When comparing rapid triage with the MTS, there was 20.9% disagreement in categorizing patients’ priority, the first being more inclusive for high priority, as shown in Table 1.

**Table 1:** Distribution of the visits (n=182), according to severity categories (high or low priority) identified by rapid triage and by the Manchester Triage System (MTS). São Paulo, SP, Brazil, 2017

Rapid triage	Manchester Triage System		
	High priority n (%)	Low priority n (%)	Total n (%)
High priority	124 (68.1%)	30 (16.5%)	154 (84.6%)
Low priority	8 (4.4%)	20 (11.0%)	28 (15.4%)
Total	132 (72.5%)	50 (27.5%)	182 (100.0%)

As previously described, it was necessary to test some variables, since the gold standard for rapid triage performed at the emergency entrance is not clearly defined. In the analysis of the performance of the two systems in the prediction of the different variables tested (Table 2), rapid triage showed better sensitivity and worse specificity than the MTS. In addition, the NPVs were better than the PPVs, showing that, when classifying patients as low priority, both rapid triage and the MTS portray a lesser probability of them presenting the given condition, that is, staying for more than 24 hours in the hospital, being admitted to the emergency observation unit or to the ICU, and/or evolve to death.

**Table 2:** Performance of rapid triage (RT) and of the Manchester Triage System (MTS) in predicting the length of hospital stay, admission to the emergency observation unit, admission to the intensive care unit (ICU), and death variables. São Paulo, SP, Brazil, 2017

Variables	Time of hospital stay over 24 hours		Admission to the emergency observation unit		Admission to the Intensive Care Unit		Death	
	RT^||^	MTS^¶^	RT^||^	MTS^¶^	RT^||^	MTS^¶^	R^T||^	MTS^¶^
**Sensitivity**	88.1%	79.7%	94.1%	83.3%	88.9%	55.6%	95.8%	91.7%
**Specificity**	17.1%	30.9%	27.5%	41.3%	15.6%	26.6%	17.1%	30.4%
**PPV** *	33.8%	35.6%	62.3%	64.4%	5.2%	3.8%	14.9%	16.7%
**NPV** ^†^	75.0%	76.0%	78.6%	66.0%	96.4%	92.0%	96.4%	96.0%
**PLR** ^‡^	1.1	1.2	1.3	1.4	1.1	0.8	1.2	1.3
**NLR** ^§^	0.7	0.7	0.2	0.4	0.7	1.7	0.2	0.3
**Accuracy**	40.1%	46.7%	64.8%	64.8%	19.2%	28.0%	27.5%	38.5%

* PPV = Positive predictive Value;

† NPV = Negative predictive value;

‡ PLR = Positive likelihood ratio;

§ NLR = Negative likelihood ratio;

|| RT = Rapid triage;

¶ MTS=Manchester Triage System

For the admission in the emergency observation unit variable, the best performance values of the systems were identified in comparison to the other variables studied, especially by rapid triage. The values of PPV and PLR in the prediction of admission to the emergency observation unit were quite similar between the systems (Table 2). However, when analyzing the results of the NPVs, it is noted that the probability of non-severe patients being admitted to the emergency observation unit was lower by rapid triage (100.0% - 78.6% = 21.4%) than by the MTS (100.0% - 66.0% = 34.0%) and this is due to the good NLR associated with the low priority classification by rapid triage. It is also noteworthy that rapid triage showed a lower rate of undertriage and a higher rate of overtriage than the MTS in all the scenarios evaluated.

## Discussion

Triage is essential for any health service, especially in places where emergency overcrowding is part of the routine of the professionals. Some special situations, such as the performance of rapid triage by nurses to assess patients with spontaneous demand, are necessary, considering the specificities of each service. Thus, knowing the performance of this rapid triage is an important step towards targeting improvement strategies in the early identification of critically ill patients who arrive at the emergency entrance and in the organization of care flows, in order to increase the survival of this population.

With regard to the characteristics of the patients evaluated, the higher frequency of females corroborates with the majority of the findings of several studies carried out in emergency services^(^ 1 ^,^ 8 ^-^ 13 ^)^, while the mean age identified was higher than in the results of other research^(^ 1 ^,^ 4 ^,^ 8 ^-^ 11 ^)^.

Among the previous comorbidities presented by the patients, researchers who analyzed the performance of the MTS in a population of adults^(^ 2 ^)^ also identified the prevalence of systemic arterial hypertension. The high frequency of comorbidities presented by the patients seems to reflect the predominance of the medical clinic as responsible for the first medical care in the series. In addition, the advanced age of the research patients may also have contributed to this predominance of clinical care. A Korean study^(^ 12 ^)^ that evaluated the complaints of older adults on arrival at the emergency showed that 80.7% of the consultations were related to the clinical disease associated with the patient’s comorbidity and only 18.5% to the occurrence of acute conditions.

The main outpatient diagnosis included the category of symptoms, signs and abnormal findings of clinical and laboratory exams, not elsewhere classified, a result that differs from the study carried out in Switzerland that identified, in the analysis of 2,407 patients admitted to the emergency department, that the main diagnoses were related to neurological (26.4%) and cardiovascular (25.2%)^(^ 2 ^)^.

The mortality rate in the sample was substantially higher than those found in the literature^(^ 8 ^,^ 14 ^)^. However, it is worth noting that the sample was composed only of patients who reported severity when they arrived at the emergency service and, therefore, were potentially more serious than the general population seeking ED, frequently investigated in other studies.

The occurrence of a death among those who were screened as a low priority by nurses during the rapid triage is highlighted. For this case, the MTS was classified as orange and the patient died after 18 days of hospitalization. Deaths like this, considered unexpected or preventable, should be evaluated for possible causes, to identify whether or not there was a failure in the triage and/or treatment process.

When comparing rapid triage with the MTS, it was possible to notice agreement in 68.1% of the visits classified as high priority, that is, those patients were in a serious condition from the perspective of the two prioritization method. Rapid triage, however, was more inclusive for high priority and some hypotheses can be raised: the possible fear of the triage nurse to underestimate the severity of the patient and/or the presence of anxiety or pressure from family members for immediate medical care. A number of studies reinforce these assumptions by identifying that the greatest difficulties encountered by professionals in triage are the population’s lack of knowledge about the classification system and the importance of different levels of priority^(^ 15 ^)^, as well as the professional’s discomfort in the face of the patient/family’s suffering who, sometimes, out of empathy, attributes greater severity to the patient during triage to speed up care and shorten such suffering/anguish^(^ 16 ^)^. There is also the insecurity of the professionals in relation to the possibility of deterioration of the clinical condition of the patient awaiting care and the tensions arising from hostile acts by patients and/or family members.

As for the performance of the two systems, rapid triage was more sensitive and less specific than the MTS in all the variables analyzed. It is difficult to say which level of sensitivity or specificity is acceptable to conclude that a given triage system is safe since, to achieve high sensitivity (i.e., an acceptable degree of undertriage), specificity must be so low that the potential to save resources would be insignificant^(^ 17 ^)^.

Regarding the length of hospital stay, the PPVs of the two systems are considered low. This can be justified by the effectiveness of the treatment performed and, thus, by the shorter hospital stay. As an example: if treated efficiently and quickly, a moderate or severe asthma attack (patient classified as high priority) can be solved promptly, not requiring a hospital stay longer than 24 hours. Or even a patient with a decrease in the level of consciousness due to hypoglycemia, after the intravenous correction of blood glucose levels, can in most cases be discharged from hospital only with the guidance of the team.

Thus, there are patients at high risk for clinical deterioration and who, if cared for in a timely manner, will be discharged in less than 24 hours - and, in some cases, will not even be admitted to the emergency observation unit. A Brazilian study^(^ 18 ^)^ reinforces the high frequency of patients who seek assistance in the emergency service due to the decompensation of chronic diseases, such as hypertensive, asthmatic and hypoglycemic crises, often reversed during the initial care in the emergency room.

Regarding admission to the ICU, although the MTS has shown greater accuracy in relation to rapid triage, although the accuracy of both is low, the MTS values relevant to the PLR (0.8) and NLR (1.7) are contradictory. It is understood that patients classified as high priority by this system were less likely to be admitted to the ICU, and patients classified as low priority were more likely to be admitted to the critical unit. The MTS also had a 2.2% undertriage rate, and almost half of the patients who were admitted to the ICU were classified as low priority. Thus, the probability that a patient classified as high priority being admitted to the ICU was only 3.8% (PPV) by the MTS. Two assumptions can be raised to explain this finding: the MTS performs poorly to correctly identify patients in need of intensive care, and the limited number of ICU beds (n=12) in the study institution may have resulted in patient allocation in the ED for treatment. A multicenter study^(^ 19 ^)^ carried out in Europe found, in absolute numbers, that the MTS classifies 14% to 20% of the adults who need ICU admission as low priority, indicating that system improvement is still necessary.

In the analysis of the performance of rapid triage in the prediction of admission in the emergency observation unit, this system presented a higher rate of overtriage and a lower rate of undertriage than the MTS. For the MTS, these values were better than that found in a study that analyzed 900 trauma victims admitted to the ED^(^ 18 ^)^.

In this context, the inclusion of patients who are not really in a serious condition in the high priority category (overtriage) can lead to overcrowding in the critical care area and impair work dynamics, in addition to unnecessarily using resources^(^ 2 ^)^. In cases of undertriage (inclusion of critically ill patients at low priority), there is a longer time between the patient’s arrival at the emergency service and the first medical care, which may result in the patient’s clinical worsening and in a worse prognosis^(^ 2 ^)^. An American study^(^ 20 ^)^ that analyzed 50,576 patients identified a significant number of patients who waited for more than 10 minutes for the triage routine, and the researchers reinforce the possible impact of this delay on the quality of care provided.

The NPVs for both systems were better than the PPVs, demonstrating that the systems were more assertive in assigning low priority than high priority for the admission in the emergency observation unit variable. In addition, patients who were classified as low priority by rapid triage (NLR = 0.2) were less likely to be admitted to the emergency observation unit than those classified as low priority by the MTS (NLR = 0.4). In this sense, it can be said that rapid triage performed by nurses at the emergency entrance performed better than the MTS in predicting the non-admission of low priority patients in the emergency observation unit.

As for the death outcome, the two systems showed similar values in most of the analyses performed. Researchers who investigated the triage given by an institutional protocol with the outcomes of patients seen at an emergency unit in the inland of São Paulo identified that, among the patients considered severe, the death rate corresponded to 66.7% and, in the group of low priority of care, this rate was 1.7%^(^ 14 ^)^. There was also a group of patients who were not classified by the institutional protocol due to situations of extreme severity and, therefore, were referred directly to the emergency room, where they had a death rate of 31.4%^(^ 14 ^)^. Another study identified that patients classified as high priority for care by the MTS have a 5.58 times greater chance of progressing to death than those classified as low priority^(^ 8 ^)^. In the present study, both rapid triage and the MTS performed better in predicting non-death for patients in the low priority group (NLR = 0.2 and 0.3, respectively) than in predicting death for patients in the high priority group (PLR = 1.2 and 1.3, respectively).

Thus, it is possible to assert that the best values of the performance tests of the two systems (rapid triage and MTS) were identified in the prediction of the patient’s admission in the emergency observation unit, among all the analyzed variables. This finding may be related to the characteristics of the service (for example: low availability of ICU beds, unit dynamics, etc.) and of the treatment (for example: quality of care, excellence of the professionals, etc.), which possibly had an influence on other outcomes and/or variables analyzed.

It is essential that managers and professionals working in emergency services understand the impact that undertriage or overtriage can have on the work dynamics and/or on the clinical evolution of the patients. Therefore, constant evaluation of the processes related to triage should be the object of attention and investigation by these professionals with the aim of improving the care flows and, consequently, optimizing resources and guaranteeing the quality of the care provided to the patient who seeks the emergency service.

Finally, the present study did not intend to indicate the best triage instrument (rapid triage or MTS), but rather to highlight findings that may contribute to the improvement of the triage process and of the organization of managerial and assistance flows in emergency services that speed up the access of critically ill patients to the service.

Some limitations of the research must be highlighted: the study was carried out in a single emergency center of a secondary level hospital, and this fact must be considered when generalizing the results. Furthermore, there was difficulty in identifying a reliable gold standard to assess the performance of the systems, although different variables have been tested.

## Conclusion

Rapid triage was more inclusive in identifying patients with high priority of care and performed better than the MTS in predicting admission in the emergency observation unit in the case of patients with spontaneous demand who reported severity on arrival at the emergency service.

For nurses who perform rapid triage, the classification of patients as low priority appears to be clear (less undertriage), but they still overestimate others, classifying them as high priority (more overtriage).

Therefore, the results of this study can contribute to the organization of managerial and care flows aimed at the rapid triage process performed by nurses at the emergency entrance, as well as indicate the need for more evidence on the main signs and symptoms that reflect the real severity of the patients, contributing to the reduction of overtriage, to the optimization of the use of resources, and to safety in the classification of patients.
